# Nitrite Oxidizer Activity and Community Are More Responsive Than Their Abundance to Ammonium-Based Fertilizer in an Agricultural Soil

**DOI:** 10.3389/fmicb.2020.01736

**Published:** 2020-08-04

**Authors:** Yang Ouyang, Jeanette M. Norton

**Affiliations:** ^1^Department of Plants, Soils and Climate, Utah State University, Logan, UT, United States; ^2^Department of Microbiology and Plant Biology, Institute for Environmental Genomics, University of Oklahoma, Norman, OK, United States

**Keywords:** nitrifying community, nitrite oxidizing bacteria, nitrogen fertilizer, *nxrB*, *Nitrospira*, potential nitrite oxidation

## Abstract

Autotrophic nitrification is mediated by ammonia oxidizing bacteria (AOB) or ammonia oxidizing archaea (AOA) and nitrite oxidizing bacteria (NOB). Mounting studies have examined the impact of nitrogen (N) fertilization on the dynamic and diversity of AOA and AOB, while we have limited information on the response of the activity, abundance, and diversity of NOB to N fertilization. We investigated the influence of organic and inorganic N fertilizers on soil NOB in silage corn field plots that received contrasting nitrogen (N) treatments: control (no additional N), ammonium sulfate (AS 100 and 200 kg N ha^−1^), and compost (200 kg N ha^−1^). Nitrifying community was examined using a universal marker (16S rRNA gene), functional gene markers (AOB *amoA* and *Nitrospira nxrB*), and metagenomics. The overall nitrifying community was not altered after the first fertilization but was significantly shifted by 4-year repeated application of ammonium fertilizers. *Nitrospira* were the dominant NOB (>99.7%) in our agricultural soil. Both community compositions of AOB and *Nitrospira* were significantly changed by ammonium fertilizers but not by compost after 4 years of repeated applications. All nitrifiers, including comammox, were recovered in soil metagenomes based on a gene-targeted assembly, but their sequence counts were very low. Although N treatment did not affect the abundance of *Nitrospira nxrB* determined by real-time quantitative PCR, ammonium fertilizers significantly promoted rates of potential nitrite oxidation determined at 0.15 mM nitrite in soil slurries. Understanding the response of both ammonia oxidizers and nitrite oxidizers to N fertilization may initiate or improve strategies for mitigating potential environmental impacts of nitrate production in agricultural ecosystems.

## Introduction

Nitrification, the oxidation of ammonium to nitrite and nitrate, mobilizes soil nitrogen (N), and promotes N loss through nitrate leaching and denitrification and, therefore, often reduces N use efficiency in agricultural ecosystems (Norton and Ouyang, 2019). Autotrophic nitrification has generally thought to be a two-step process: ammonia oxidation to nitrite by ammonia oxidizing bacteria (AOB) and ammonia oxidizing archaea (AOA) and nitrite oxidation to nitrate by nitrite oxidizing bacteria (NOB). Remarkably, recent research has uncovered that some *Nitrospira*, a diverse and widespread known NOB, are able to convert ammonia to nitrate within one organism in the complete oxidation of ammonia to nitrate known as comammox (Daims et al., 2015; van Kessel et al., 2015). Since nitrifiers are generally slow growing and recalcitrant to isolation, culture-independent molecular techniques, targeting 16S ribosomal RNA (rRNA) or functional marker genes, have been primarily used to investigate the quantity and diversity of these organisms in natural and managed ecosystems (Rotthauwe et al., 1997; Purkhold et al., 2000; Leininger et al., 2006; Attard et al., 2010; Prosser, 2011; Pester et al., 2012, 2014). High throughput sequencing of 16S rRNA genes detects the diversity and richness of all nitrifying groups in a whole microbial community without using specific functional markers (Purkhold et al., 2000; Zhou et al., 2015). The *amoA* gene, which encodes the alpha subunit of ammonia monooxygenase, is a frequently and widely used functional marker gene for ammonia oxidizers (Rotthauwe et al., 1997; Leininger et al., 2006; Pjevac et al., 2017). The genes encoding the alpha or beta subunit of nitrite oxidoreductase (*nxrA* or *nxrB*) have been developed to detect and quantify NOB in pure cultures and environmental samples (Poly et al., 2008; Pester et al., 2014). Functional marker genes of nitrifiers, such as *amoA* and *nxrB*, often provide a higher phylogenetic resolution than 16S rRNA for discriminating nitrifiers on the strain and species levels (Norton et al., 2002; Pester et al., 2014, Aigle et al., 2019).

Nitrogen fertilization is an important management practice for crop production that exerts a significant influence on the nitrifying community in agricultural systems. Several research groups have documented that AOB are more responsive than AOA to N fertilization in agricultural soils (Jia and Conrad, 2009; Taylor et al., 2012; Carey et al., 2016; Ouyang et al., 2016, 2018). Although the response of ammonia oxidizers to N fertilization has been extensively examined, we have limited information about the ecology of NOB in agricultural systems. The NOB belong to seven genera in the phyla of *Proteobacteria*, *Nitrospirae*, *Nitrospinae*, and *Choloroflexi* (Daims et al., 2016). The genera *Nitrobacter* and *Nitrospira* are considered as the two main players in soils (Poly et al., 2008; Wertz et al., 2008; Attard et al., 2010). Previous studies found that *Nitrobacter* spp. were more responsive than *Nitrospira* spp. to tillage practices (Attard et al., 2010), organic amendments (Ollivier et al., 2013; Han et al., 2018), and N fertilization (Han et al., 2018).

In a previous study, we assessed temporal changes in the abundance and diversity of AOA and AOB, using *amoA* as marker gene, under organic and conventional N management, and showed that the abundance and community of AOB, but not AOA, were significantly shifted by 3-year repeated mineral N fertilization (Ouyang et al., 2016). The objective of the current study was to complement our previous examination using 16S rRNA gene marker in the same field. A more commonly used primer set (amoA1F/2R) was applied to re-examine the diversity of AOB *amoA* using high-throughput sequencing. We expected to find the similar results as our previous study. Since our 16S rRNA gene-based high throughput sequencing revealed that *Nitrospira* were the dominant nitrite oxidizers (>99.7%) recovered in our soil, a newly developed gene marker, *nxrB* of *Nitrospira*, was used to expand our understanding of the abundance and diversity of nitrite oxidizers under contrasting N treatment. Understanding the response of both ammonia oxidizers and nitrite oxidizers to N fertilization may initiate or improve strategies for mitigating potential environmental impacts of nitrate production in agricultural ecosystems.

## Materials and Methods

### Soil Characterization

The details of the agricultural site (North Logan, Utah, USA), experimental design, treatments, soil sampling, and soil characteristics have been previously described (Ouyang et al., 2016, 2017). The soil is an irrigated, very strongly calcareous Millville silt loam (Coarse-silty, carbonatic, mesic Typic Haploxeroll) with a pH of approximately 8.0. The experimental design is a randomized complete block with four blocks and four nitrogen treatments: control (no N fertilization), ammonium sulfate (AS 100 and 200 kg N ha^−1^), and steer-waste compost (200 kg total N ha^−1^). Treatments were surface applied in May of each year and incorporated by tilling immediately after application. Silage corn was planted within 2 days and irrigation was applied according to general agricultural practice in the area.

### Potential Nitrite Oxidation

We measured potential nitrite oxidation (PNO) for soils sampled in 2015. PNO was determined using a modified method of Wertz et al. (2007). Samples of 3 g of fresh soil were placed in a 100 ml Erlenmeyer flask, and then 30 ml of a solution of NaNO_2_ (final 0.15 mM NaNO_2_) was added. Preliminary experiments measured PNO with a range of final nitrite concentrations (0.05, 0.1, 0.15, 0.3, and 1 mM) and showed that there was no nitrite inhibition and enough nitrite left after 24 h with 0.15 mM nitrite. Flasks were covered with foil with small hole and incubated at 30°C for 24 h with shaking (200 rpm). After incubation for 1, 6, 20, and 24 h, 1.5 ml of the suspension was sampled using pipette with a wide mouth tip, and centrifuged (10,000 *g*) for 5 min. Supernatants were then transferred to a new tube and nitrite concentration in the supernatants was analyzed spectrophotometrically using the Griess reagent (Mulvaney, 1996). The change in nitrite was used to calculate the linear rate of nitrite consumption over the 24 h period.

### Soil DNA Extraction and Real-Time Quantitative PCR

Soil DNA was extracted following the MoBio Power Soil DNA isolation protocol (MoBio Laboratories Inc., Carlsbad, USA) using 0.25 g moist soil. DNA extracts were quantified by using a NanoDrop spectrophotometer (NanoDrop Technologies, Wilmington, DE). Quantitative PCR (qPCR) of *Nitrospira nxrB* gene was performed using the SsoAdvanced SYBR Green Supermix and a CFX CONNECT real-time PCR detection system (Bio-Rad laboratories, Hercules, CA, USA). Primers, nxrB169R and nxrB638F, were used to quantify *Nitrospira nxrB* gene abundance (Pester et al., 2014). Standard curve was constructed with plasmids containing cloned *nxrB* products from environmental DNA. The 25 μl reaction mixture contained 12.5 μl SsoAdvanced SYBR Green Supermix (Bio-Rad laboratories, Hercules, CA, USA), 0.5 μM of both reverse and forward primers, and 10 μl of 10-fold diluted soil DNA extract. Amplifications were carried out as follows: an initial denaturation step of 95°C for 10 min, 40 cycles of 95°C for 45 s, 56.2°C for 1 min, and 72°C for 45 s, and a final extension step of 72°C for 10 min. Fluorescence intensity was read during the 72°C step of each cycle. Reaction efficiencies ranged from 94 to 105%, and *R*^2^ values ranged from 0.992 to 0.999.

### Illumina Sequencing and Data Analysis for Bacterial *amoA* and *Nitrospira nxrB*

Sequencing of the bacterial *amoA* and *Nitrospira nxrB* amplicon libraries was accomplished for soils sampled in June 2014. The primer set (amoA1F/2R) was used to examine the diversity of AOB *amoA* (Rotthauwe et al., 1997). The same *Nitrospira nxrB* primers described above were used for high-throughput sequencing. The preparation and Miseq sequencing were the same as our previous study (Ouyang and Norton, 2020). Raw forward and reverse reads were merged using the USEARCH workflow (Edgar, 2013). High quality *amoA* and *nxrB* sequences were extracted from merged reads in each sample using the RDP SeqFilters with a read Q score cutoff of 20 (Cole et al., 2014). The obtained sequences were further processed using the FrameBot tool (Wang et al., 2013) to fix frame shifts. Sequences were dereplicated and clustered at 90% nucleotide similarity using USEARCH. Operational taxonomic unit (OTU) table and taxonomy file were organized for diversity analysis using R package phyloseq (McMurdie and Holmes, 2013). A neighbor-joining phylogenetic tree was constructed from top 20 OTUs using a Kimur 2-parameter distance with 1,000 bootstrap replicates with MEGA 6 (Tamura et al., 2013).

### Soil Metagenome Processing and Gene Targeted Assembly

Metagenomes were also obtained from soils samples in June 2014 (Ouyang and Norton, 2020). DNA samples from four replicates of each N treatment were pooled with equal amount of DNA. DNA were then sequenced on the Illumina HiSeq 2,500 platform with 2 × 150 bp paired-end format, processed at the Joint Genome Institute (JGI) and data are available at the IMG/M website (Chen et al., 2017). Gene-targeted assembly was conducted for the quality-filtered metagenomes using Xander (Wang et al., 2015). Five genes involved in nitrification (AOA *amoA*, AOB *amoA*, comammox *amoA*, *Nitrobacter nxrB*, and *Nitrospira nxrB*) and *rplB* (encoding ribosomal protein L2) were included for the assembly. For each gene of interest, seed sequences, hidden Markov models (HMMs), and nucleotide and protein reference sequences were downloaded from FunGene (Fish et al., 2013). Default assembly parameters were used and sequences were clustered at 95% amino acid similarity. A representative sequence from each cluster was searched against the reference gene database and the nonredundant database (nr) from NCBI using BLAST (Camacho et al., 2009). Some sequences of *nxrB* were removed if they were not a match with *Nitrospira* or *Nitrobacter*.

### Identify Nitrifying Community With 16S Illumina Sequencing

The detailed information about Illumina sequencing with 16S rRNA gene and bioinformatics analysis was described in Ouyang and Norton (2020). Soil nitrifying community, including AOA, AOB, and NOB, was evaluated by screening 16S rRNA gene sequences. The relative abundance of AOA, AOB, NOB, and their specific OTUs were calculated by dividing the total 16S rRNA sequences in each sample. The OTU representatives of NOB were selected to create a phylogenetic tree, which was constructed by neighbor-joining using a Kimur 2-parameter distance with 1,000 bootstrap replicates with MEGA 6 (Tamura et al., 2013).

### Statistical Analysis

Statistical analysis of the effect of N treatment and year on AOA, AOB, NOB, and selected OTU relative abundances was completed using two-way analysis of variance (ANOVA) with the Proc Mixed model in SAS 9.2 (SAS Institute, Inc., Cary, NC, USA). Treatment and year were used as fixed effects and block as a random effect. One-way ANOVA was used to the effect of N treatment on the relative abundance of *amoA* and *nxrB* top 20 OTUs. Data were log transformed as necessary to meet normality assumptions. Repeated measures ANOVA was used to analyze effects of treatment on *nxrB* copy numbers and PNO rates in soils sampled in 2015. Any values of *p* ≤ 0.05 were considered to be statistically significant. The Bray-Curtis distance matrix was created by using the relative abundance of all OTUs from nitrifying community. To carry out the beta-diversity analysis, the AOB *amoA* and *Nitrospira nxrB* libraries were first normalized to 432 and 380 reads, respectively (fewest sequences per sample). The rarefaction curves showed that these normalized reads were sufficient to capture the diversity of amoA and nxrB. Nonmetric multidimensional scaling (NMDS) and permutational multivariate ANOVA (PerMANOVA) were conducted to visualize and assess the Bray-Curtis distance matrix in *vegan* package of R software^1^.

## Results

### The Activity and Abundance of *Nitrospira*

**Figure 1:**
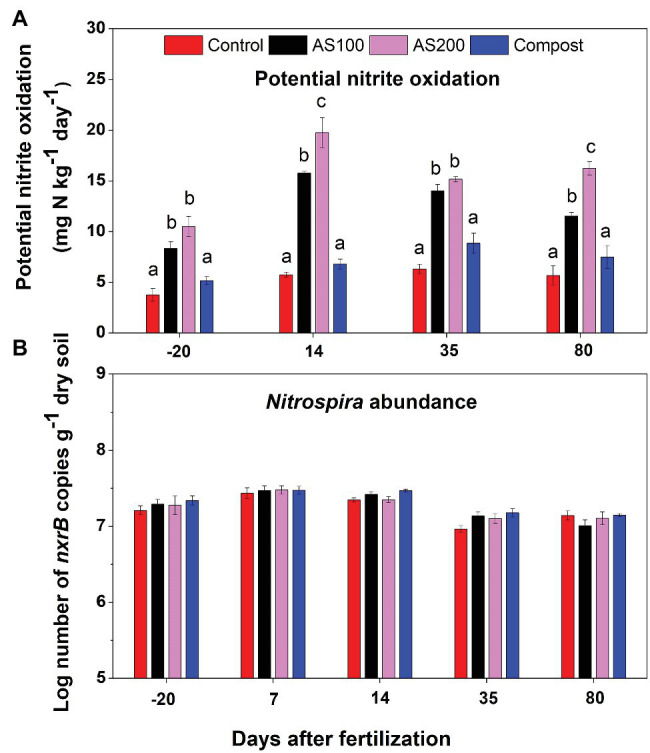
**(A)** Nitrite oxidation potentials in 2015 before and after fertilization across four Nitrogen (N) treatments [control (no N fertilization), ammonium sulfate (AS 100 and 200 kg N ha^−1^), and compost (200 kg N ha^−1^)]. Nitrite oxidation potential was measured by soil slurry assay supplemented with 0.15 mM NO_2_^−^. Error bars represent standard errors (*n* = 4). Different letters above the bars indicate a significant difference among treatments within a specific sampling time (*p* < 0.05) based on repeated measures ANOVA. **(B)** Abundance of *Nitrospira nxrB* gene copy numbers (log_10_ transformed) across four N treatments. Error bars represent standard errors (*n* = 4).

PNO rates ranged from 4 to 20 mg N kg^−1^ d^−1^. Repeated ANOVA analysis indicated that both treatment (*F* = 116.72, *p* < 0.0001) and sampling time (*F* = 116.55, *p* < 0.0001) significantly influenced PNO rates, and there was a treatment and sampling time interaction effect (*F* = 11.86, *p* < 0.001). PNO rates were significantly increased by AS treatments, but unchanged by compost, compared with control treatment (Figure 1A). PNO rates were lowest for soils sampled before fertilization. In addition, PNO rates were relative higher for soils sampled 14 days after fertilization compared with other sampling times in both AS treatments. *Nitrospira nxrB* copy numbers, ranging from 7.94 × 10^6^ to 2.95 × 10^7^ per gram of dry soils, showed no treatment effect (*F* = 1.66, *p* = 0.24), but has significant time effect (*F* = 72.70, *p* < 0.001). *Nitrospira nxrB* copy numbers were highest on 7 days after fertilization across all N treatments (Figure 1B). *Nitrospira nxrB* copy numbers were not significantly correlated with PNO rates (*r* = 0.02, *p* = 0.89).

### Nitrifying Community Composition Recovered in 16S rRNA Gene Illumina Sequencing

**Figure 2:**
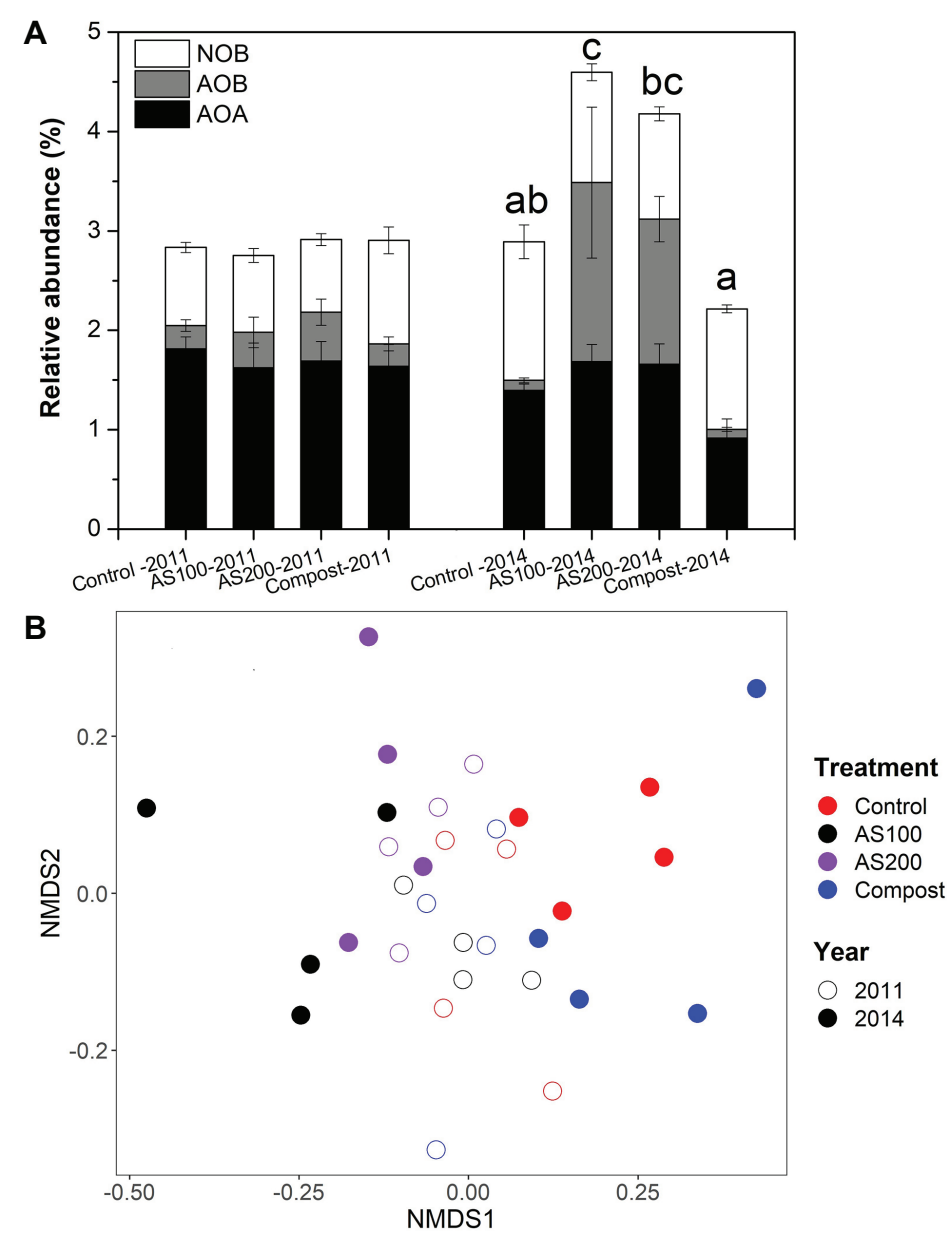
**(A)** Relative abundance of nitrifying populations based on differences in 16S rRNA genes for four N treatments. Error bars represent standard errors (*n* = 4). Different letters above the bars indicate a significant different in total nitrifying populations in 2014. Abbreviation: AOA, ammonia oxidizing archaea; AOB, ammonia oxidizing bacteria; NOB, nitrite oxidizing bacteria. **(B)** Nonmetric multidimensional scaling (NMDS) ordination based on the abundance of 16S rRNA gene operational taxonomic units (OTUs) of all nitrifying populations. Colors denote treatment (red = control, black = AS100, purple = AS200, blue = compost). Fill denotes sampling time (open = 2011, solid = 2014).

The structure of nitrifying community was examined by screening 16S rRNA gene sequences of AOA, AOB, and NOB for Aug 2011 and Jun 2014 samples. We obtained 30,405, 11,181, and 19,236 sequence reads for AOA, AOB, and NOB, respectively, with 19, 8, and 22 unique OTUs, respectively. We only obtained one OTU, which grouped with *Nitrobacter*. *Nitrospira* were the dominant nitrite oxidizers (>99.7%) recovered in all soil samples. Soil nitrifiers represent 3–5% of total prokaryotic community. The relative abundance of soil nitrifiers was not changed by N treatment in 2011, but their relative abundance was significantly increased by AS treatments but decreased by compost treatment in 2014 (Figure 2A and Supplementary Table S1). Specifically, in 2014, AOB proportion was strongly increased by AS treatments, and AOA proportion was significantly reduced by compost treatment, but NOB proportion was unchanged by N treatments (Figure 2A).

Nitrifying community structures were different between 2011 and 2014, and N treatment strongly changed nitrifying community in 2014 (Figure 2B). Two-way PerMANOVA confirmed that nitrifying community composition was significantly affected by year (*p* < 0.001), treatment (*p* < 0.001), and their interaction (*p* = 0.002). Seven OTUs were selected for detailed statistical analysis that had relative abundances higher than 0.1% of total prokaryotic community. None of those seven OTUs showed a N treatment effect in 2011 (Supplementary Figure S1 and Supplementary Table S1). The relative abundance of AOB OTU 23, related to *Nitrosospira multiformis*, was increased significantly by AS treatments in 2014. Three selected AOA OTUs, belonged to the *Nitrososphaera* cluster, were reduced by compost treatment in 2014. *Nitrospira* OTU_13 and OTU_276 belonged to Lineage II, but OTU_202 belongs to Lineage V (Supplementary Figure S2). The relative abundance of *Nitrospira* OTU_13 was decreased by all N fertilizers and OTU_202 was increased by AS treatment while OTU_276 was increased by compost treatment in 2014.

### Diversity of Bacterial *amoA* and *Nitrospira nxrB*

The structure of AOB and *Nitrospira* communities was also evaluated using Illumina high-throughput sequencing analysis of *amoA* and *nxrB* genes, respectively. A total of 22,980 and 27,061 high-quality raw sequence reads were obtained for AOB and *Nitrospira*, respectively, with 44 and 70 unique OTUs, respectively (90% identity cut off). One of the samples from control treatment was removed due to low reads (<150).

**Figure 3:**
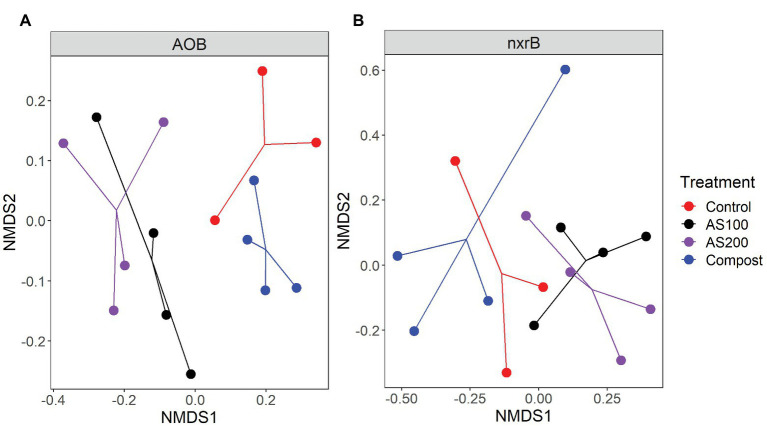
NNMDS ordination based on the Bray-Curtis distance matrices showing the change in AOB **(A)** and *Nitrospira* **(B)** community composition for four N treatments.

**Figure 4:**
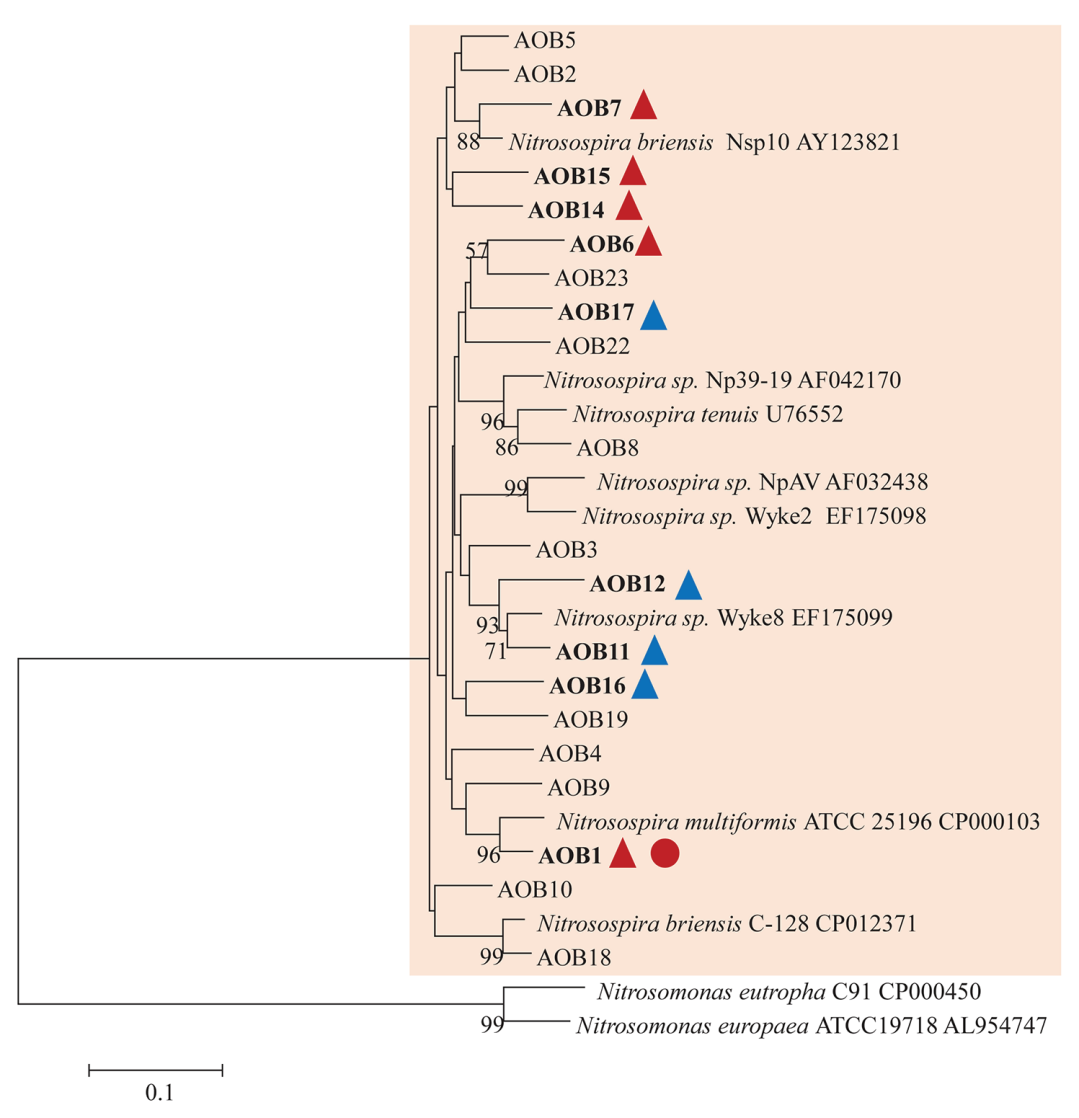
Neighbor joining tree for AOB partial *amoA* top 20 OTUs (covered 98.2% of total sequences). The scale bar represents 10% nucleic acid sequence divergence, and bootstrap values (>50%) are showed at branch points. OTUs are shown in bold if they were significantly changed by N treatment. Up triangles indicate a significant difference between values for control and AS treatments while circles indicate a significant difference between values for control and compost treatments. Red color indicates a significant higher abundance than control while blue color indicates a lower abundance.

AOB community structure was significantly affected by N treatment (*R*^2^ = 0.47, *p* = 0.002). Pairwise comparison indicated that there was no difference between control and compost treatments. However, AS treatments were significantly different from control and compost treatments (Figure 3A). Phylogenetic analysis of top 20 abundant OTUs (contributing 98.2% of total sequences) showed that these abundant OTUs were all affiliated with the *Nitrosospira* Cluster 3 lineage (Figure 4). Nine abundant OTUs were significantly changed by N treatment (ANOVA, *p* < 0.05). Specifically, AOB1 (closely related to *Nitrosospira multifomis* ATCC 25196) was significantly higher in N fertilized treatments than control treatments (Figure 6A). AOB6, AOB7, AOB14, and AOB15, which were all closely related to *Nitrosospira briensis* Nsp10, were significantly higher in AS treatments than control and compost treatments. However, AOB11, AOB12, AOB16, and AOB17, which were all closely related to *Nitrosospira* sp. Wyke8, were significantly higher in control and compost treatments than in AS treatments.

**Figure 5:**
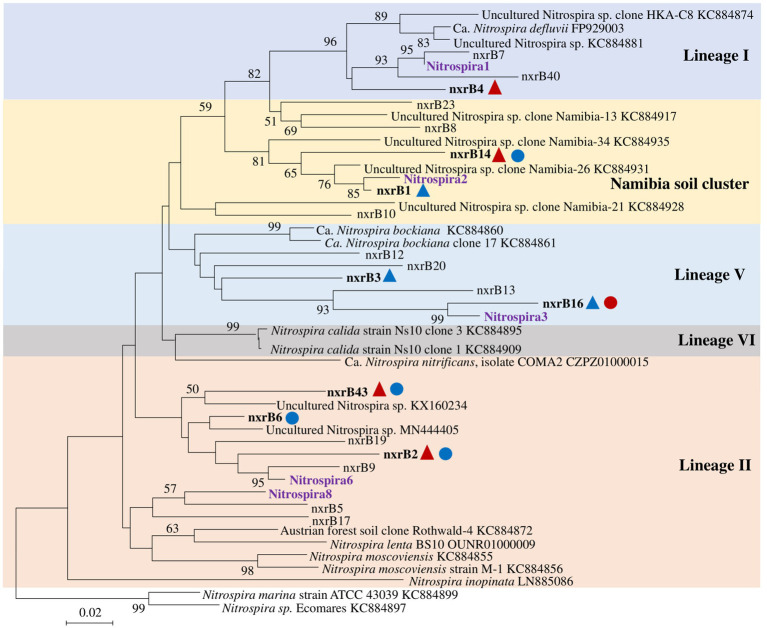
Neighbor joining tree for *Nitrospira* partial *nxrB* top 20 OTUs (covered 94.9% of total sequences). The scale bar represents 1% nucleic acid sequence divergence, and bootstrap values (>50%) are showed at branch points. OTUs are shown in bold if they were significantly changed by N treatment. Up triangles indicate a significant difference between values for control and AS treatments, while circles indicate a significant difference between values of control and compost treatments. Red color indicates a significant higher abundance than control treatment, while blue color indicates a lower abundance.

**Figure 6:**
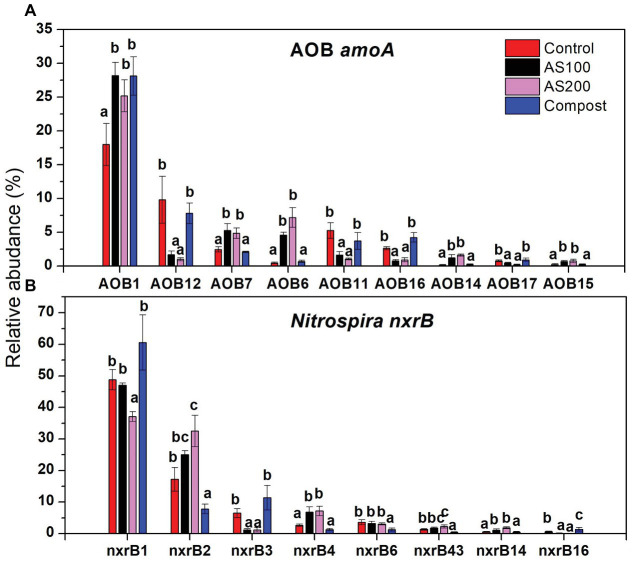
Relative abundances of selected AOB **(A)** and *Nitrospira* **(B)** top 20 OTUs, which were significantly changed by N treatments. Error bars represent standard errors (*n* = 4). Different lowercase letters indicate significant differences in relative abundance among the N treatments within an OTU (*p* < 0.05).

*Nitrospira* community structure was also significantly affected by N treatment (*R*^2^ = 0.55, *p* = 0.002). Similarly, pairwise comparison indicated that there was no difference between control and compost treatments. However, AS treatments were significantly different from control and compost treatments (Figure 3B). Phylogenetic analysis indicated that those top 20 abundant OTUs (contributing 94.9% of total sequence OTUs) were affiliated with at least five different *Nitrospira* lineages (Figure 5). Eight abundant OTUs were significantly changed by N treatment (Figure 6B; ANOVA, *p* < 0.05). For example, in compost treatment, nxrB16 (lineage V) was significantly higher than in control treatment, while nxrB2 and nxrB4, nxrB43, and nxrB6, which belonged to lineage II, were significantly lower. In AS treatments, nxrB2, nxrB4, nxrB43 and nxrB14 were significantly higher than control treatments, while nxrB1, nxrB3, and nxrB16 were significantly lower.

### Gene-Targeted Assembly for Nitrifiers

Five selected nitrification genes were all recovered in soil metagenomes based on gene-targeted assembly (Supplementary Table S2). The number of OTUs recovered at 95% amino acid identity for these nitrification genes ranged from 2 to 8, and their rplB normalized abundances ranged from 0 to 3.8% in four soil metagenomes. There were six OTUs for AOA, belonging to *Nitrososphaera* and *Nitrosocosmicus* (Supplementary Table S3). There were seven OTUs for AOB and most of them were closely related to *Nitrosospira*. AS treatments had relatively higher AOB abundance than control and compost treatments. Interestingly, two comammox OTUs were recovered in our metagenomes with very high similarity (>98%) to current available comammox genome bins (Supplementary Table S3, *Nitrospira* sp. SG-bin1). *Nitrospira* were recovered in all four metagenomes, while *Nitrobacter* were only present in AS treatments.

## Discussion

### Ammonium-Based Fertilizer Changed the Activity and Community Composition, but Not the Abundance of *Nitrospira*

Ammonia oxidation has generally been considered the rate-limiting step of nitrification. Recent studies also showed that PNO was much higher than potential ammonia oxidation (Ke et al., 2013). However, compared to our nitrification potentials (NP), PNO rates were much lower than NPs in the same soil samples (Ouyang et al., 2017). We also measured the dynamic of nitrite during the NP assays for soils sampled in Aug 2015. The result showed that nitrite accumulated linearly during the 24 h shaking-period, with the proportion increasing from 6 to 43% (Supplementary Figure S3). Taken together, our observation suggested that nitrite oxidation is the rate-limiting step during the NP assay with non-limiting ammonium availability and continuous shaking. It is noteworthy that the accumulation of nitrite during the NP assay may not be representative of field conditions. Ammonium availability is limited for most of the growing season in field soils causing ammonia oxidation rates to be constrained by diffusion to the cell surface in the soil matrix (Stark and Firestone, 1995).

We observed that PNO was increased by ammonium-based fertilization but not by compost fertilization. The high nitrite concentration from ammonia oxidation shortly after ammonium-based fertilization may stimulate the enzyme production of nitrite oxidoreductase. However, *nxrB* copy numbers were not changed by either ammonium-based fertilization or compost. One of the reasons may be that the genomes of *Nitrospira* often contains multiple copies of *nxrB*, ranging from 2 to 6 copies in currently available *Nitrospira* genomes (Pester et al., 2014). Therefore, detection of *nxrB* copies may neither reflect actual changes in the abundance of *Nitrospira* nor the actual enzyme. In our study, PNO was not correlated with *nxrB* gene copy numbers. Several previous studies reported that PNO was strongly and significantly correlated with the abundance of *Nitrobacter*-like NOB but weakly correlated with the abundance of *Nitrospira*-like NOB (Attard et al., 2010; Han et al., 2018). Although *Nitrobacter* were recovered in 16S and metagenomic analysis in our study, their relative abundance was extremely low compared to that of *Nitrospira*. Ongoing investigations will use qPCR and high-throughput amplicon sequencing to further examine any contribution of *Nitrobacter* to nitrite oxidation in our soils.

Based on the high-throughput sequencing analysis of *Nitrospira nxrB*, *Nitrospira* community composition was significantly changed by ammonium-based fertilizers but not organic fertilizers. However, Han et al. (2018) showed that both inorganic and organic fertilization significantly influenced the *Nitrospira* community composition. Manure, with relatively higher N availability than the compost used in our case, was continuously applied for 26 years in their study. We might also observe effects of organic fertilization if followed over decades. Interestingly, at the OTU level, abundant OTUs could be both increased and decreased by organic and inorganic N fertilization. The pattern was relatively consistent to each specific lineage. The variety of patterns of *Nitrospira* OTUs in response to N fertilization indicates that the *Nitrospira* community is physiologically diverse in their response (Daims et al., 2016). We have been conducting an enrichment of this NOB experiment in our lab, detailed investigation at the single cell level may provide new insight in the response of NOB to N availability.

Most described comammox belong to *Nitrospira* lineage II (Daims et al., 2015; van Kessel et al., 2015). Similar *Nitrospira* were recovered from three of four of our soil metagenomes. We could not examine the response of comammox to N fertilization using the limited sequences recovered from soil metagenomes.

### Comparison of Molecular Techniques for Soil Nitrifiers

Illumina sequencing targeting 16S rRNA genes provided comparable numbers of sequence reads to pyrosequencing of *amoA* genes performed in our earlier study (Ouyang et al., 2016), suggesting that high-throughput sequencing of 16S rRNA genes is powerful enough to detect the nitrifying community, even though they are a small fraction (2–5%) of the total microbial community. Therefore, nitrifying community can be examined using the same universal primers targeting the 16S rRNA genes when accomplished with a very high sequencing depth. However, it is noteworthy that the 16S rRNA gene has low phylogenetic resolution for some closely related nitrifiers. For example, phylogenetic analysis of AOB *amoA* indicated 20 or 44 different OTUs from *Nitrosospira*, while 16S rRNA gene showed only one AOB OTU which belongs to *Nitrosospira*. In comparisons of full-length *amoA* genes with 16S rRNA genes, *Nitrosospira multiformis* ATCC 25196 and *Nitrosospira multiformis* 24-C showed 97% identity in the 16S rRNA gene sequence but approximately 88% similarity in the full-length *amoA* gene (Norton et al., 2002).

High-throughput sequencing of 16S rRNA genes enabled us to examine the changes in relative frequencies of AOA, AOB, and NOB in response to N treatment. Similar to our previous work, AOB was more responsive than AOA to ammonium fertilizers after 4 years of repeated application (Ouyang et al., 2016). In addition, it is interesting to observe the reduction of relative abundance of AOA in compost treatment, possibly due to the increase of other members of the microbial community, such as *proteobacteria* (Ouyang and Norton, 2020). Different primers for *amoA* are often used to examine the abundance and diversity of ammonia oxidizers (Meinhardt et al., 2015; Ouyang et al., 2018). We got similar community-level and species-level result with a more commonly used primer set (amoA1F/2R) for the diversity of AOB *amoA* compared with our previous study (Ouyang et al., 2016). Specifically, the overall AOB community was significantly changed by ammonium fertilizers. Several OTUs closely affiliated with *Nitrosospira* sp. Wyke8 were reduced by AS treatments suggestive that this group may be inhibited by high ammonium concentrations as found previously (Webster et al., 2005). Our study suggests that the selection of primers had relatively limited impact on the analysis of AOB community.

Shotgun metagenomics has been previously used to examine the diversity of soil nitrifiers (Wang et al., 2015; Orellana et al., 2018). However, this approach has a limitation to the detection of low-abundance genes of interest, such as genes involved in nitrification, in complex soil samples (Rodriguez-R et al., 2018). In our study, we only recovered 2–8 OTUs for nitrifiers in metagenomes with relative high sequence depth (>33 Gb). With few counts in each OTU, we were unable to assess relative abundance by this method. Therefore, the nitrifiers recovered from soil metagenomes have low coverage and provide a relative weak statistical power for the comparison of treatment or temporal dynamics. However, soil metagenomes may provide the preliminary data for the discovery of novel organisms or functions. For example, even though we recovered limited sequences putatively related to comammox and *Nitrobacter*, this information confirmed the presence of those two nitrifiers and stimulated their further detailed examination.

## Conclusion

Our investigation of nitrifying community using 16S rRNA gene marker complemented previous reports using the *amoA* gene marker, indicating that 4-year repeated N fertilization but not one time N fertilization significantly change the relative abundance and composition of the nitrifying community. Inorganic N fertilizers strongly stimulated the rates of potential nitrite oxidation. N fertilizers, regardless of inorganic or organic, had no effect on the abundance of *nxrB* of *Nitrospira*, although the abundance of *nxrB* showed significant temporal variation. Nitrite oxidizers were found to be dominated by *Nitrospira* by 16S rRNA sequencing. Their community composition as indicated by *Nitrospira* targeted *nxrB* genes was shifted by ammonium-based fertilization.

## Data Availability Statement

The datasets presented in this study can be found in online repositories. The names of the repository/repositories and accession number(s) can be found at: https://www.ncbi.nlm.nih.gov/, SRP109290, SRP109299, SRP109302, SRP109289. MiSeq amplicon reads may be accessed from PRJNA597781.

## Author Contributions

JN designed the study and revised the manuscript. YO conducted the experiment, analyzed the data, and wrote the draft of the manuscript. All authors have read and approved the final manuscript.

### Conflict of Interest

The authors declare that the research was conducted in the absence of any commercial or financial relationships that could be construed as a potential conflict of interest.

## Footnotes

^1^http://www.r-project.org
