# Supplementary material for: Nitrite Oxidizer Activity and Community Are More Responsive Than Their Abundance to Ammonium-Based Fertilizer in an Agricultural Soil
Source: Front Microbiol. 2020 Aug 4;11:1736. doi: 10.3389/fmicb.2020.01736 (PMC7417772; doi:10.3389/fmicb.2020.01736)

Table S1. Results of the two-way ANOVA of the effect of N sources and year on the relative abundance of selected 16S rRNA OTUs (nitrifying populations).

| Factors | AOB | | AOA | | NOB | | OTU 23 | | OTU 55 | | OTU 67 | | OTU 80 | | OTU 13 | | OTU 202 | | OTU 276 | |
|---|---|---|---|---|---|---|---|---|---|---|---|---|---|---|---|---|---|---|---|---|
| | F | P | F | P | F | P | F | P | F | P | F | P | F | P | F | P | F | P | F | P |
| Treatment | 5.94 | *** | 2.02 | ns | 3.00 | ** | 4.2 | ** | 0.8 | ns | 4.34 | ** | 0.99 | ns | 3.96 | ** | 4.15 | ** | 7.29 | *** |
| Year | 6.76 | ** | 4.47 | ** | 29.28 | *** | 4.51 | ** | 0.93 | ns | 2.59 | ns | 11.14 | *** | 32.88 | *** | 0.51 | ns | 4.15 | ** |
| Treatment* Year | 3.77 | ** | 1.91 | ns | 1.81 | ns | 2.5 | ns | 0.97 | ns | 2.13 | ns | 0.96 | ns | 3.71 | ** | 3.53 | ** | 1.8 | ns |

Asterisks highlight significant P values (*** P < 0.01, ** P< 0.05).

Table S2 Xander assembly of nitrification genes in soil metagenomes.

| Sample | Control | AS100 | AS200 | Compost |
|---|---|---|---|---|
| File size (GB) | 33 | 39 | 43 | 76 |
| # AOA_OTUs | 3 | 4 | 5 | 4 |
| # AOB_OTUs | 1 | 5 | 5 | 3 |
| # Comammox_OTUs | 1 | 0 | 1 | 1 |
| # Nitrospira_OTUs | 3 | 5 | 6 | 9 |
| # Nitrobacter_OTUs | 0 | 2 | 2 | 0 |
| AOA_abundance[a] | 3.50% | 2.50% | 3.80% | 2.20% |
| AOB_abundance[a] | 0.50% | 2.70% | 2.30% | 0.60% |
| Comammox_abundance[a] | 0.20% | 0 | 0.20% | 0.20% |
| Nitrospira_abundance[a] | 1.76% | 1.53% | 1.17% | 1.52% |
| Nitrobacter_abundance[a] | 0.00% | 0.38% | 0.30% | 0.00% |

[a] The abundance for nitrification genes was normalized to total abundance of the *rplB* gene.

Table S3. Counts for protein OTUs of nitrification functional genes by N treatment in June 2014 metagenomes. Best match of protein OTUs to the NCBI databases shown.

| OTUs | Control | AS100 | AS200 | Compost | Length (aa) | Accession# | Identify (%) | Organism(s) |
|---|---|---|---|---|---|---|---|---|
| AOA1 | 8 | 4 | 8 | 12 | 211 | WP_148681392 | 96.88 | *Candidatus Nitrososphaera gargensis* |
| AOA2 | 3 | 3 | 6 | 4 | 211 | WP_148681392 | 96.21 | *Candidatus Nitrososphaera gargensis* |
| AOA3 | 3 | 4 | 6 | 4 | 211 | WP_144731401 | 97.16 | *Candidatus Nitrosocosmicus arcticus* |
| AOA4 | 0 | 0 | 0 | 2 | 102 | WP_134482982 | 95.10 | *Candidatus Nitrosocosmicus* (multi-species) |
| AOA5 | 0 | 2 | 2 | 0 | 211 | WP_134482982 | 94.31 | *Candidatus Nitrosocosmicus* (multi-species) |
| AOA6 | 0 | 0 | 1 | 0 | 107 | WP_144731401 | 100.00 | *Candidatus Nitrosocosmicus arcticus* |
| AOB1 | 2 | 5 | 8 | 4 | 273 | WP_074703664 | 98.17 | *Nitrosospira multiformis* |
| AOB2 | 0 | 3 | 1 | 1 | 273 | WP_090371561 | 97.44 | *Nitrosospira sp. Nl5* |
| AOB3 | 0 | 2 | 2 | 0 | 273 | WP_107694664 | 96.34 | *Nitrosospira sp. Nsp2* |
| AOB4 | 0 | 2 | 2 | 0 | 273 | WP_097065283 | 99.63 | *Nitrosospira sp.* (multi-species) |
| AOB5 | 0 | 2 | 0 | 0 | 151 | WP_074798855 | 94.70 | *Nitrosospira briensis* |
| AOB6 | 0 | 0 | 0 | 1 | 107 | WP_090415883 | 98.13 | *Nitrosomonas halophila* |
| AOB8 | 0 | 0 | 1 | 0 | 119 | WP_074703664 | 94.96 | *Nitrosospira multiformis* |
| Comammox1 | 1 | 0 | 1 | 0 | 244 | OQW38018 | 98.36 | *Nitrospira sp. SG-bin1* |
| Comammox2 | 0 | 0 | 0 | 2 | 101 | TKB71914.1 | 100.00 | *Nitrospira sp.* |
| Nitrospira1 | 5 | 6 | 5 | 8 | 429 | WP_053381688 | 98.60 | *Nitrospira moscoviensis* |
| Nitrospira2 | 1 | 1 | 0 | 2 | 429 | WP_053381688 | 97.90 | *Nitrospira moscoviensis* |
| Nitrospira3 | 0 | 0 | 0 | 2 | 301 | WP_062483507 | 96.01 | *Candidatus Nitrospira inopinata* |
| Nitrospira6 | 0 | 1 | 1 | 0 | 242 | WP_090900834 | 94.63 | *Candidatus Nitrospira nitrificans* |
| Nitrospira8 | 0 | 0 | 0 | 2 | 254 | WP_090900834 | 94.49 | *Candidatus Nitrospira nitrificans* |
| Nitrospira12 | 1 | 0 | 0 | 0 | 140 | WP_053381688 | 90.71 | *Nitrospira moscoviensis* |
| Nitrospira13 | 0 | 0 | 1 | 0 | 118 | WP_053381688 | 90.68 | *Nitrospira moscoviensis* |
| Nitrospira14 | 0 | 0 | 0 | 1 | 118 | WP_053381276 | 92.37 | *Nitrospira moscoviensis* |
| Nitrobacter1 | 0 | 2 | 1 | 0 | 140 | WP_141385421 | 100.00 | *Nitrobacter winogradskyi* |
| Nitrobacter3 | 0 | 0 | 1 | 0 | 125 | WP_079447846 | 98.40 | *Nitrobacter vulgaris* |

FIGURE S1. The relative abundance of partial 16S rRNA OTUs of nitrifying populations (relative abundance > 0.1%) among four N treatments in Aug-2011 (**A**) and Jun-2014 (**B**) soil samples. Error bars represent standard errors (n=4). Different lowercases above the bars indicate a significant difference among treatments in a specific year (p<0.05), based on two-way ANOVA.

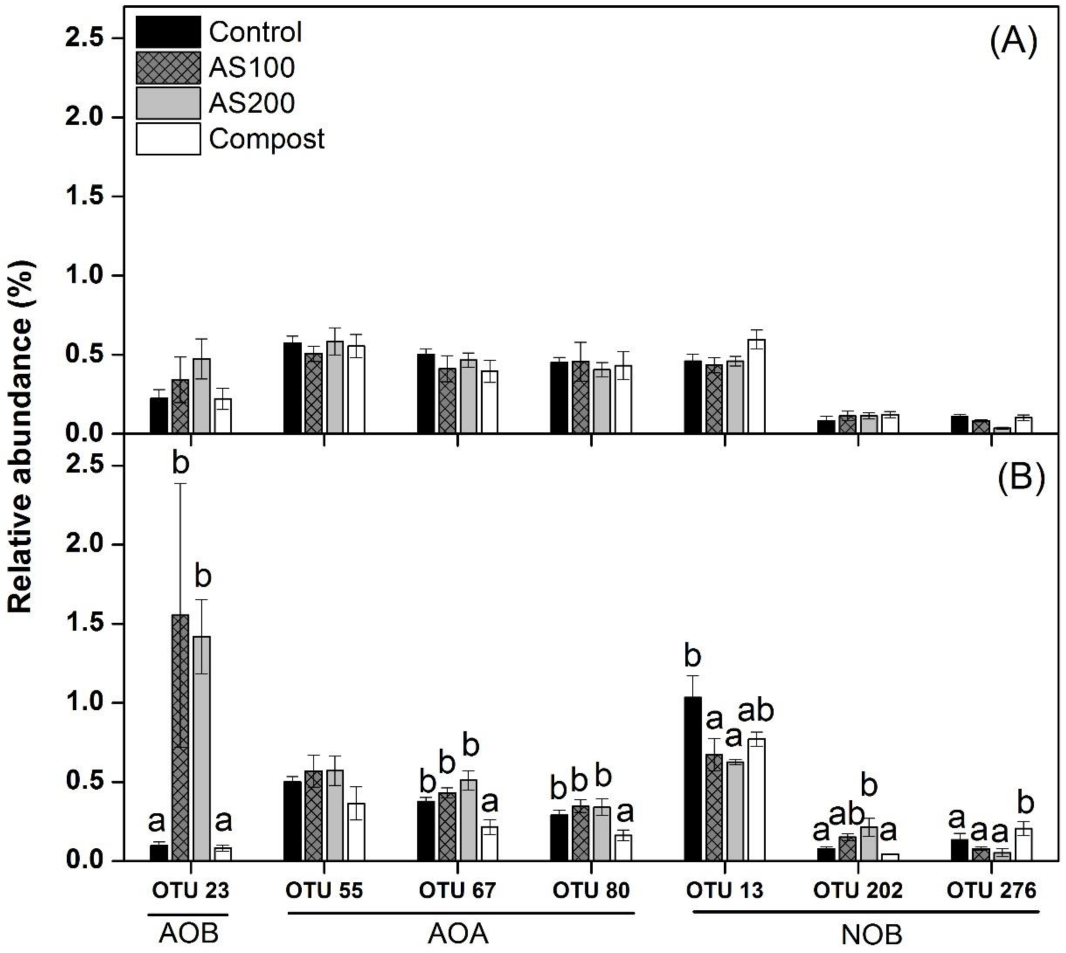

FIGURE S2. Neighbor joining tree for *Nitrospira* 16S OTUs based on a 253 bp nucleotide sequence alignment. The scale bar represents 1% nucleic acid sequence divergence, and bootstrap values (>50%) are showed at branch points. OTUs are shown in bold if significantly changed by N treatment. Up triangles indicate a significant difference between values for controls and AS treatments, while circles indicate a significant difference between values for controls and compost treatment. Red color indicates a significant higher abundance than control, while blue color indicates a lower abundance.

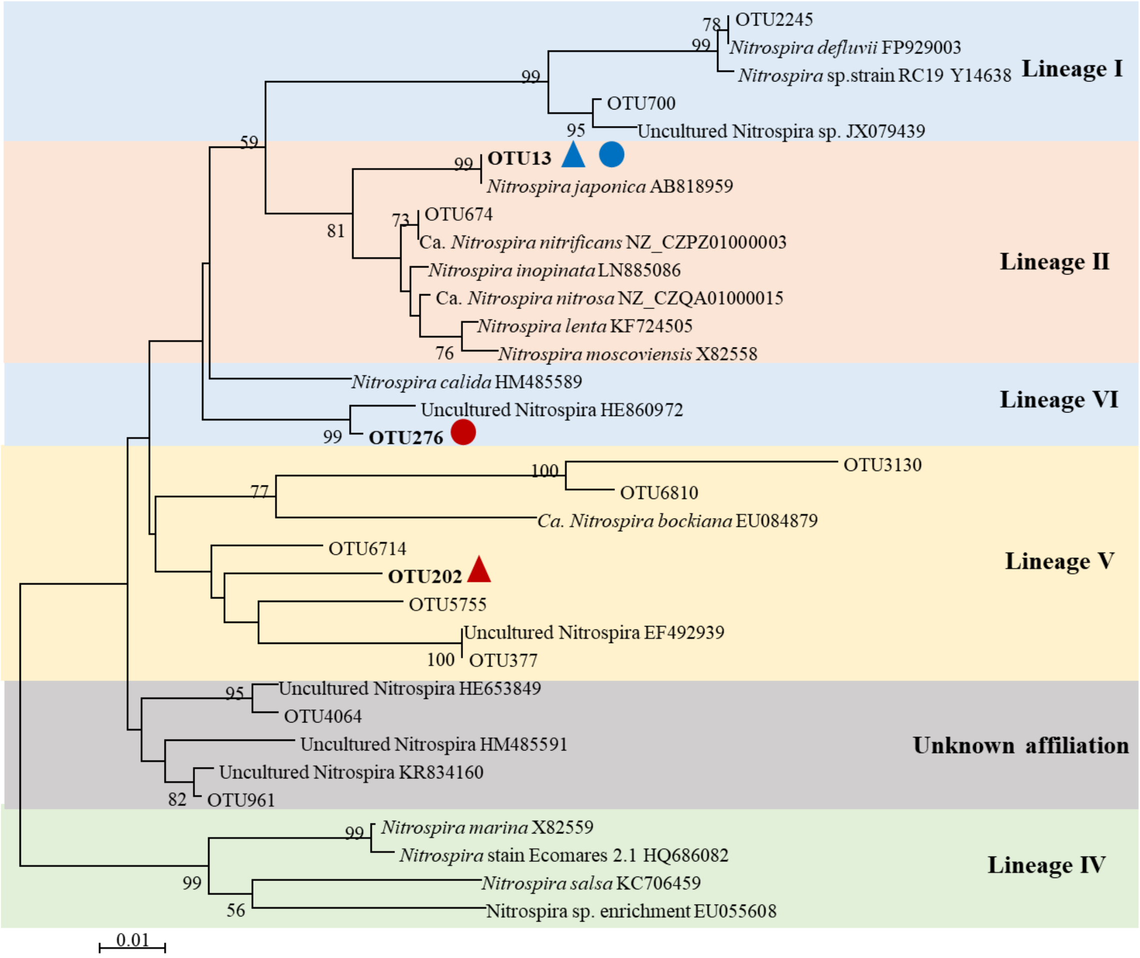

FIGURE S3. Changes in nitrite and nitrate concentration during the nitrification potential assay measured by soil slurry supplemented with 1 mM $NH_4^+$ at 30 °C. Note scale difference for AS treated soils. Error bars represent standard error (n=4).

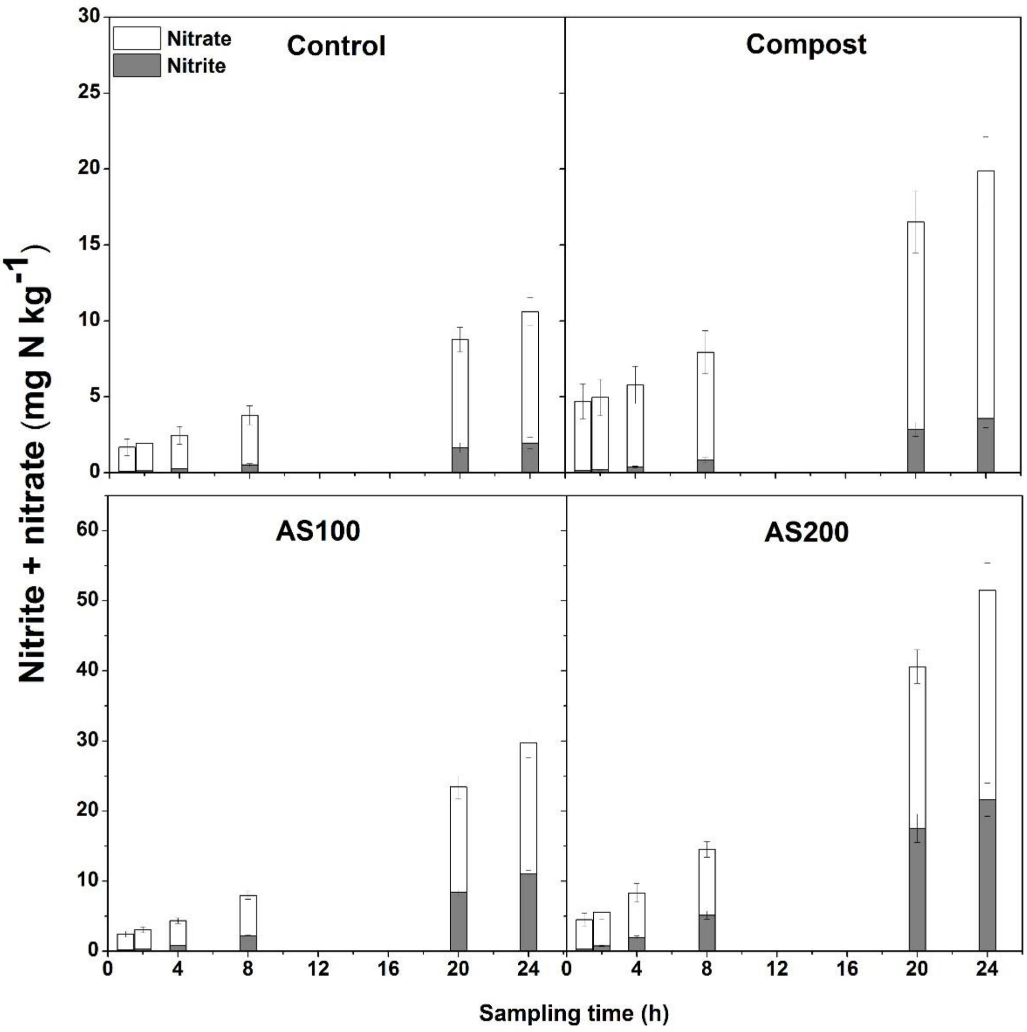

Supplement: Supplementary file 1 [file Data_Sheet_1.PDF]
